# Are Malaria Risk Factors Based on Gender? A Mixed-Methods Survey in an Urban Setting in Ghana

**DOI:** 10.3390/tropicalmed6030161

**Published:** 2021-09-02

**Authors:** Virginia Quaresima, Tsiri Agbenyega, Bismark Oppong, Julia Ann D. A. Awunyo, Priscilla Adu Adomah, Eunice Enty, Francesco Donato, Francesco Castelli

**Affiliations:** 1 University Department of Infectious and Tropical Diseases, University of Brescia and ASST Spedali Civili of Brescia, 25123 Brescia, Italy; francesco.castelli@unibs.it; 2 Department of Civil, Environmental, Architectural Engineering and Mathematics (DICATAM), University of Brescia, 25123 Brescia, Italy; 3 Centro di Ricerca Emato-Oncologica AIL (CREA), Diagnostic Department, ASST Spedali Civili of Brescia, 25123 Brescia, Italy; 4 Kwame Nkrumah University of Science and Technology (KNUST), Kumasi, Ghana; tsiri@ghana.com; 5 HopeXchange Medical Centre, Kumasi, Ghana; ppongb5@gmail.com (B.O.); awunyojulia28@gmail.com (J.A.D.A.A.); jayjese2@gmail.com (P.A.A.); adwoaenty@yahoo.com (E.E.); 6 University Department of Hygiene, Epidemiology and Public Health, University of Brescia, 25123 Brescia, Italy; francesco.donato@unibs.it; 7 UNESCO Chair ‘Training and Empowering Human Resources for Health Development in Resource-Limited Countries’, University of Brescia, 25123 Brescia, Italy

**Keywords:** malaria, gender, preventive measures, exposure behaviors, insecticide-treated nets

## Abstract

Malaria still represents one of the most debilitating and deadly diseases in the world. It has been suggested that malaria has different impacts on women and men due to both social and biological factors. A gender perspective is therefore important to understand how to eliminate malaria. This study aimed to investigate malaria from a gender perspective in a non-for-profit private health facility, HopeXchange Medical Centre, based in Kumasi (Ghana). A sequential mixed-methods design, comprising quantitative and qualitative methods, was used. This study found low ownership (40%) and use (19%) of insecticide-treated nets (ITNs). Most malaria cases were women (62%), who were less educated and had more external risk factors associated with infection. Our study reported a trend of preferring malaria self-medication at home, which was practiced mostly by men (43%). Our data suggest that women are more likely to be exposed to malaria infections than men, especially due to their prolonged exposure to mosquito bites during the most dangerous hours. Our study highlighted the need for future malaria control policies to be more focused on social and behavioral aspects and from a gender perspective.

## 1. Introduction

Malaria is a debilitating and deadly disease that still causes 409,000 deaths every year and results in significant suffering and human misery, predominantly in sub-Saharan-Africa [1]. As documented in the World Malaria Report 2020, in 2019, out of 29 countries that account for 95% of malaria cases globally, 27 are in sub-Saharan Africa. Sixty-seven percent of deaths due to malaria occur among children under the age of 5 years [1]. Although 9 million fewer malaria cases were registered in 2019 than in 2000 (respectively, 229 million and 238 million malaria cases) significant progress in reducing global malaria cases have not been made since 2014.

In the World Malaria Report 2019, Ghana has been listed along with Nigeria, among the countries with the highest increase in cases of malaria in 2018 (8% increase, 0.5 million more cases) [2]. To support countries in pursuing the global technical strategy for malaria 2016–2030 (GTS) milestones, the World Health Organization (WHO) and Roll Back Malaria (RBM) have proposed the high-burden-to-high-impact (HBHI) approach to be implemented in the 11 countries with the highest burden of malaria, and Ghana is among them [3].

It has been suggested that malaria has a different impact on women than men due to social and biological factors [4]. The term *gender* refers to the economic, social and cultural attributes and opportunities associated with being male and female, whereas the term *sex* refers to male and female differences in immunological, anatomical and physiological differences that influence exposure, clearance and susceptibility to infections. Gender relations thus define how women and men at all ages organize their lives in all aspects, including duties, responsibilities, and opportunities. A previous study conducted in Ghana highlighted the importance of approaching malaria management from a gender perspective. This includes looking within the household at how the social and economic power of women and men can influence decisions to respond to illness [5].

Along with a gender perspective, the risk of malaria infection intrinsically depends on the surrounding settings. Indeed, it has been documented how the risk of malaria in urban and rural areas markedly differs; urban malaria transmission is widely heterogenous and closely related to clusters of people living near parks, water, and small-scale agriculture lands [6,7]. In Ghana, as in many other West African countries, the rapid urban growth and the transformation of agricultural into urban land have deeply influenced the local livelihoods, negatively affecting the environment and health [8]. As cities expand physically, the frontiers between urban, peri-urban and rural activity are more undefined and could merge. Peri-urban areas are those areas surrounding cities which are in most ways integrated with the city.

In such a complex scenario, the combined use of both quantitative and qualitative methods in low-middle-income countries (LMICs) has been recognized as having the most highly urbanized setting [9]. Noteworthy, in recent years two methodologies have been often combined in public health research, generally referred to as “mixed methods”. Quantitative and qualitative research can be considered complementary, methodologically. Qualitative research is based on observation, all different aspects of a reality that are socially constructed and, hence, subjectively interpreted. In contrast, quantitative research is usually based on observable and measurable reality that can be quantified and objectively interpreted. Qualitative research can provide additional understanding about a research topic that may be inaccessible using quantitative methods.

This study is based on a combined approach of qualitative and quantitative methods, to investigate malaria from a gender perspective in a highly urbanized setting in Ghana, where urban malaria is not fully understood or comprehensively investigated.

## 2. Aims

Our study aimed to investigate malaria from a gender perspective at HopeXchange Medical Centre (HXC), a non-for-profit private health facility based in Kumasi (Ghana) and included in the state-funded Christian Health Association of Ghana (CHAG).

The main objectives of the study were:

1. To ascertain whether sociodemographic characteristics, clinical symptoms, and laboratory parameters of *P. falciparum* malaria in adult cases differ by sex;
2. To analyze the factors influencing differences *in P. falciparum* malaria awareness, exposure behaviors, and preventive measures in adult cases according, to sex.
3. A secondary objective was to investigate malaria treatment-seeking behaviors within households with regard to gender dynamics, using qualitative research methods.

## 3. Methods

### 3.1. Study Site and Patients

This study was conducted during the 2018 rainy season (between 1 June and 22 October) at the HXC, located in the suburbs of Kumasi (Ghana), which is holoendemic for *P. falciparum* malaria, and is characterized by a perennial transmission and seasonal peaks (high transmission from May to October); the principal malaria vectors of this area are mosquitoes of the *Anopheles gambiae* complex and *Anopheles funestus* [10].

Ecologically speaking, Ghana can be divided in three ecological zones: coastal, forest and northern savanna, which differ by climate and general environmental characteristics. Kumasi is located in the forest zone and it is part of the Ashanti region, which is one of the 10 administrative regions of Ghana. Kumasi is the second largest city of the country, and the health facility where this study was conducted is part of the district named Kumasi Metropolitan Assembly (KMA) and of the Bantama Sub-metro. KMA ranks second as the municipality with the highest number of poor persons in the country (88,935 poor persons) [11]. The submetro is organized in 81 communities and 35 community-based health planning and services (CHPS). The HXC is part of the Nzema CHPS and specifically in the Nwamase Community with 5200 inhabitants in 2017.

The HXC catchment area includes urban villages such as Fankyenembra, Odeneho Kwadaso, Santasi, Aburaso, as well as peri-urban villages such as Darko, Hemang, Apire, Nwamase, and Ampatia.

The study population comprised a consecutive sample of recruited adults (≥18 years) presenting at HXC OPD during the study period with symptoms suggestive of malaria, who had resided for the last 30 days in the same household (defined as residence units sharing a common internal patio, as it is the usual living compound arrangement in this area). Pregnant women were ineligible. The diagnosis was confirmed by the Malaria Rapid Diagnostic Test (RDT) and microscopy. The severity of the disease was analyzed according to the WHO definitions [12] based on symptoms and selected laboratory parameters (glycaemia [Gly], parasite density [PD], hemoglobin [Hb] values). 

### 3.2. Quantitative Methods

#### 3.2.1. Prospective Assessment of Malaria Cases in Adults

The characteristics of laboratory-confirmed *P. falciparum* malaria cases in adults were assessed. Blood samples (finger prick or venous blood) for thick films and malaria RDT were collected from the patients at entry, and parasite density (asexual parasites/µL) was determined by trained laboratory technicians. Two types of RDTs were used: MalariAg P.f™ test kit (SD Bioline, Cat. No. 05FK50), based on the qualitative detection of *P. falciparum* histidine-rich protein II (HRP-II) antigen; and Malaria AgPf (HRP-2/pLDH) RDT (SD Bioline, Cat. No. 05FK90), with *P. falciparum* double antigens (HRP-2 and lactate dehydrogenase, LDH).

Throughout the study both RDTs were employed, following the HXC Laboratory Malaria Diagnostic Algorithm. Hb values were assessed by the semi-automatic device Cell Dyn Ruby Hematology analyzer, (Roche, Basel, Switzerland) or, in case of shortage of reagents, a point-of-care portable device Hemocue 201+™ (Hemocue, Ängelholm, Sweden). Gly was measured by the portable device Statstrip Xpress Glucometer (Nova Biomedical Corporation, Waltham, MA, USA).

#### 3.2.2. Evaluation of Sociodemographic Characteristics, Awareness, Exposure Behaviors and Preventive Measures in Malaria Cases

Malaria awareness, exposure behaviors and preventive measures were assessed using a specific questionnaire based on malaria toolkit materials [13] and other previous studies conducted in Africa, and specifically in Ghana [5,14,15,16]. The questionnaire addressed: (i) composition of the household; (ii) literacy; (iii) occupation, with reference to malaria exposure risk; (iv) availability of bed nets in the household and their use, (v) general malaria preventive measures; (vi) attitude towards self-initiated medication; (vii) the household tasks which might lead to exposure to mosquito bites, and the decision-making process when needing to access health services were also investigated; along with (viii) household composition; (ix) house building materials; (x) water sanitation services; (xi) general economic status; and (xii) different mosquito breeding habitats. 

Hospital staff attended a pre-test training for questionnaire administration prior to the study period. A research nurse, a community health nurse and a health promoter were designated as research field workers (RFWs) and were trained and oriented on enrollment procedures and questionnaire administration. The RFWs administered the questionnaire in the local language (*Twi*). The malaria patients to whom the questionnaire was not administered were excluded from the study.

#### 3.2.3. Focus Group Discussions (FGDs) and In-Depth Interviews (IDIs)

A total of four focus group discussions (FGDs) [17] were conducted separately for women and men within two communities of HXC’s catchment area. Two FGDs took place in Fankyenebra (urban) and other two in Darko (peri-urban). The participants were selected following a purposive sampling, as such involving the communities who mainly access the HXC’s health service and, in both occasions, the community members were invited to join the discussion at the end of church functions. The groups were arranged in two schools upon requesting the permission to the local authorities. Each FGD ranged between five and eight consenting participants per group. RFWs conducted the discussions in *Twi*. Interviews lasted approximately 45 min each and were audio- and video-recorded. The recorded materials were translated and transcribed for subsequent analysis. Open-ended questions were used, and these were adapted over the course of FGDs. Representative quotations and statements of interest were extracted and reported to support the qualitative findings. The FGDs topics included general information about malaria, malaria first-aid, herbal drugs, and homemade preventive measures.

Fourteen in-depth Interviews (IDIs) were also conducted at HXC, including three management and administration staff, the hospital IT manager, five laboratory technicians, two nurses, one health promoter, and two medical doctors, aiming to analyze the habits and attitudes towards malaria in a group of people with higher levels of education and directly working in the health service. All IDIs were conducted in English because all interviewed staff were fluent English-speakers.

### 3.3. Statistical Analysis

Categorical variables were described as frequencies and continuous variables were expressed as mean, standard deviation (SD), and range. The comparisons of proportions between males and females were performed using the chi-square test, with a threshold of <0.05 for rejecting the null hypothesis in two-tailed tests. Computations were carried out using the STATA program for personal computer, version 14.0. (STATA Statistics/Data Analysis 12.0—STATA Corporation).

### 3.4. Ethical Considerations

Ethical approval was obtained from the Committee on Human Research, Publications and Ethics of Kwame Nkrumah University of Science and Technology (KNUST), School of Medical Sciences & Komfo Anokye Teaching Hospital (KATH). The study was conducted in compliance with recognized international standards, including the principles of the Declaration of Helsinki. The chiefs of the communities surrounding HXC were also informed. An information sheet about the study rationale and participant’s rights was provided to participants in Twi. Written consent by signature or thumb print (from those who could not write) was obtained from participants. Measures were taken to ensure privacy and respect of the dignity of all interviewees.

## 4. Results

### 4.1. Quantitative Results

#### 4.1.1. Clinical and Laboratory Features of *P. falciparum* Malaria Infection

Out of 203 adult patients (126 women and 77 men) diagnosed with malaria at HXC during the study period, 124 were included in the analysis. Seventy-nine patients were excluded because they did not meet the inclusion criteria. Patients who tested positive on RDT but negative on microscopic confirmation (n = 8), whose PD was not reported in the records (n = 11), who did not show clinical malaria symptoms (n = 3), or were pregnant (n = 3), or refuse to fill out the questionnaire (n = 54, 32 women, 22 men) were excluded.

Less than 20% of the enrolled cases were over 50 years old, approximately 61% reached the HXC from villages outside the facility catchment area, equally distributed among males and females (Table 1). Only 16 (12.9%) had not attended school, and of the 108 individuals who received an education only, 14.8% reached higher education levels. The majority of women (49.2%) had only completed primary education, while men had mostly attended up to secondary school (53.3%). Regardless of the education level achieved, significantly more females than males could not read a simple English sentence (49.4%, 21.3%, *p* = 0.007). Most of the women were traders and retailers (39% vs. 10.7%), whereas more men than women were students (men 25.5%, women 10.4%), farmers, or had other professions.

The most frequent symptom was fever (63%), followed by headache (48%), chills (36%), general malaise (26%), vomiting (23%), body-ache (14%), dizziness (11%), and diarrhea (8%), with no differences between males and females. There were no differences in the levels of Hb and Gly (Table 2) between female and male patients. Although very variable, PD did not differ in the two sexes and, in 80% of patients, fell below 10,000/µL, and 42% that ranged between 1000/µL and 10,000/µL. Patients who required infusion therapy and admission were 29 (16%).

#### 4.1.2. Gender Differences of Factors Possibly Influencing *P. falciparum* Malaria Incidence and Severity

As many as 97% of respondents answered positively to the question “Do you know what malaria is?” (Table 3). An equal proportion of men (95.7%) and women (97.4%) knew what malaria was. Likewise, malaria advertisements spread by radio, television, posters and newspapers (92%) were the most effective way of getting information. Surprisingly, only 37% of interviewees mentioned Community Health Worker (CHW) talks or hospitals (females 43%, males 28%), and schools (31%). Self-initiated medication for malaria as for other diseases, is a common practice; in this study 32% of malaria cases stated they had taken antimalarial drugs in the last month. Thirty-two out of 40 patients who self-initiated antimalarial drugs stated that they got those medications from pharmacies, shops, or markets. Self-initiation was more common in men than women (42.6% and 26%, *p* = 0.055). More than 37% of patients stated they used mosquito repellent coils, 39% usually spray indoor mosquito repellents, 54% had window screens, and 40% owned insecticide-treated nets (ITNs, 43% males and 38% females). However, about 20% of the ITN owners reported sleeping under the net “sometimes” or “never”, and 29% declared that the nets are not well maintained. Indeed, 61% of interviewees usually visit open spaces during night hours for any household activity. Regarding socializing outdoors and the need to conduct household tasks in the evening, most respondents (74%) spent time in open spaces after sunset, often for church activities, work related issues, or household tasks. In the preceding month, 50% had joined “special social gatherings” such as funerals or church events. These were generally held in open space and lasted many hours after the sunset. Significantly more women (60%) than men (37%) went into open spaces earlier than 6 am (*p* < 0.05). 

#### 4.1.3. Focus Group Discussions (FGDs) and In-Depth Interviews (IDIs)

(a) *General knowledge about malaria symptoms and transmission.*

FGDs clearly indicated that males and females were well informed about *etiridii* (Twi: “malaria”) clinical manifestations, without knowledge differences between Fankyenebra and Darko inhabitants. The majority of reported symptoms were: chills, diarrhea, joint pains, nausea, loss of appetite, fever, headache, and general body weakness. *Ntomntom* (Twi: “mosquitoes”) were identified as the sole cause of malaria, often specifically referring to the female of *Anopheles* mosquito by all the participants, but malaria infection was also attributed to smoking weed (marijuana) and alcohol abuse by the male groups. In particular one elder of Fankyenebra stated that “*if you overdrink you might get malaria*”, and some young participants of the Darko group declared that one young subject who died of malaria the week before instead died because “*he smokes too much weed*”.

Most of IDI respondents (12 out of 14) mentioned television, radio, schools, hospitals and churches as the main information sources or places where messages about malaria are spread.

(b) *Exposure and vulnerability towards malaria.*

Open drains surrounding the houses, bushy environment, bath drains, and uncovered basins for drinking and cooking water were identified by all responders as mosquito breeding sites. However, it is interesting to note the association of the spread of malaria with hygienic conditions. One female in the Fankyenebra group stated that “*Since 50 pesewas* [coins, 100 of which make 1 cedi, the currency of Ghana] *are paid for accessing the public toilet, often people defecate in plastic bags leaving them all over*”. A woman remarked that the town is full of *bola* (rubbish) everywhere. Additionally, men often referred to personal hygiene and general house cleanliness as elements linked to malarial infection. Regarding a person who died recently in Darko of severe malaria, someone said “*He got severe malaria because he was not bathing enough*”; another one stated, “*I never get malaria because I always keep the house clean*”. For Fankyenebra men “*keeping the bedroom well organized and hanging the clothes up*” reduce the risk of malaria because “*cleanliness is close to godliness*”. Finally, for one Fankyenebra woman “*Living [with] many clothes hung in the room and leaving the room messy, might foster mosquito bites*”.

Remarkably, during the urban FGDs (Fankyenebra), sickle cell disease (SCD), anemia, “red blood cell status” were mentioned as conditions leading to more serious symptoms of malaria. 

Nutrition was taken into great consideration by all women; both urban and peri-urban female linked malaria infection to oily food because “*Eating a lot of oil makes you prone to get malaria [sic]*”.

(c) *Malaria comorbidities and severe malaria.*

All responders stated that malaria is more dangerous than HIV and is a deadly disease. Only the Fankyenebra female group was aware of the potential risk of developing permanent consequences from malaria infection and they reported that “*Malaria results in convulsions and, if not treated, might also develop in psycho-motion disfunction*”. The Darko female group linked the condition of severe malaria to convulsions and a woman said that “*malaria causes injury to the brain, that’s why convulsions might occur*”. On the other hand, during the Darko discussion a man reported that “*it is not true, malaria can’t (cause disabilities). Diabetes can lead to amputation*”.

(d) *Gender-related habits and malaria.*

For the Darko male group “*The ladies get malaria more easily, because they often use the public toilet*”, while “*The majority of men don’t get malaria because they are always outside the house*”. On the other hand, for Darko females “*Men get less malaria because they wear trousers, while women’s dressing code exposes them much more to mosquito bites and consequently to malaria more often*”. The Darko female group described also some household activities generally carried out by women during late evening as further exposure to mosquito bites “*Women sit outside for a long time and they also sell goods until late in the evening, exposing themselves much more than men*”.

The nurses and the health promoter interviewed during the IDIs convincingly stated that “*Trading is one of the most common activities in the region and the tendency is to spend much time in open spaces*”; “… *when they come from work people tend to refresh outside or chat a lot before going inside*”.

Ten respondents described joining watchnight services at least once per week, and therefore being exposed in open spaces from 10 p.m. to 4 a.m. The watchnight masses are generally practiced by Christian churches and are an occasion for prolonged outdoor praying sessions.

(e) *Conventional drugs versus homemade remedies for malaria prevention and treatment.*

Malaria prevention homemade measures are orange pills known as *gari* (a powder obtained from roasted cassava flakes) which are burned on charcoal and whose smoke repels mosquitoes. Conventional preventive measures, such as mosquito nets on doors and windows, mosquito repellent spray, and mosquito coils are also used. The maintenance and use of ITNs was well known among interviewees, despite most of them being reluctant to sleep under them because of heat, claustrophobia, and skin rash. In addition, bednets do not adapt easily to the bedroom/house arrangement (square-morphology bednets are the types mainly distributed by government or non-government organization programs).

The malaria preventive measures used by IDI respondents included mostly mosquito nets on doors and windows, and indoor spraying; however bednet use is intermittent or absent. Only 3 out of 14 participants described always sleeping under the mosquito bednets. “…*people feel uncomfortable sleeping inside the net because of the heat*” and some others reported suffocating feelings. As also mentioned during the FDGs, some interviewed nurses described mosquito bednets’ shape as a problem for users, because square nets were more challenging to install in a home than the round type, which instead require only a single hook to be installed.

As described during the IDIs, *Meffee* is used as malaria repellent and consists in “*burning palm nut chaff during the night, keep mosquito away*”. Charcoal-burned lemon or orange pills were also mentioned as preventive measures to repel mosquitoes. The health promoter reported the practice of burning empty egg cartons as another method for repelling mosquitos. When feeling the early manifestations of malaria, both women and men in Darko drink much more water. One male respondent even admitted drinking his own urine as malaria treatment “*When you think you have malaria you can drink your own urine to recover. If the body doesn’t want the urine, if you take it back, anything wrong disappears*”. In Fankyenebra most respondents rely on hospitals if they suspect they have malaria, while Darko males buy medications at drugstores or pharmacy. On the contrary, females of peri-urban areas use as malaria first aid, *Taabia,* a herbal medicine sold in the market, an *enema* containing a mixture of herbs or ginger ground with chili pepper, or *Dudo*, a homemade herbal remedy made of moringa leaves and lemon leaves or pineapple skin that is brewed for drinking.

For convulsions, especially in affected children, garlic is rubbed on the body or the legs are immersed in a water pool. On the other hand, these “non-conventional” malaria treatments are rarely applied for children, who are usually immediately rushed to the hospital.

All IDIs interviewees stated that “*whenever people feel sick they first choose for self-medication from the pharmacy and going to the hospital just when the condition worsens*”; “*female are more prone to access the hospital than male, who rather prefer self-first-aid*”, although “*the work schedules are also another reason forcing males to not easily attend hospital*”. Nurses described the belief “*Men do self-medication much more than female, when they are sick they just go to the pharmacy shop… They always want to appear healthy*”.

Ten IDIs’ respondents out of 14, admitted to having used herbal drugs for treating malaria at least once in their lives. “*In Ghana the use of herbal drugs is tremendously common, especially in rural communities*”. Most respondents had been exposed throughout their lives to herbs and to herbal-medication knowledge. They were taught by family elders how to recognize and use herbs properly. Only managers and academic personnel were never exposed to such traditional practices. All respondents, however, did not use herbs sold in the market indiscriminately, without any certification or control by the Food and Drugs Authority (FDA) in Ghana. Most of the interviewees were able to mention some homemade remedies still in use. Some mentioned *neem* tree leaves or *theack* tree leaves to be brewed and drunk, another had been taught to make a mixture of leaves from mango tree, guava tree and Indian almond tree to be boiled and drunk or inhaled.

## 5. Discussion

Even though more women than men presented to hospital with malaria infection during the study period, neither parasite density nor clinical manifestations suggested gendered differences. On the other hand, this study revealed different malaria exposure behaviors and preventive measures between males and females. In addition, the mixed method approach showed the existence of economic, cultural, and social triggers that influence malaria exposure behaviors and preventive measures as well.

Infectious diseases responses require multifactorial components, including not only the availability of adequate clinical care but also improvements in the living conditions of citizens and access to education. The most vulnerable groups are likely to be affected by the dual burden of low literacy and a high prevalence of infection [18]. This study demonstrated that women with malaria have less formal education than men, as only 4% of men had not receive formal education compared to 18% of women (*p* < 0.05). This feature is in line with the Multiple Indicator Cluster Survey Six (MICS 6), conducted in 2017–2018 by Ghana Statistical Service (GSS), revealing that 19% of women didn’t attend school or pre-primary education, compared with 10% of males [19]. In addition, a higher proportion of our respondents only completed primary school (women 49%, men 29%, *p* > 0.05) with fewer having attended secondary school (women 38%, men 53%, *p* > 0.05) or reached higher education levels (women 13%, men 18%, *p* > 0.05). More females (49%) than males (21%) couldn’t read, confirming the unbalanced education distribution between genders (*p* < 0.05).

The other important gendered difference in malarial incidence is related to occupation. Indeed, significantly more female malaria patients were principally traders, while males, especially, were students from the surrounding boarding schools. This is in agreement with data reporting that, in Ashanti, 51% of the female population are engaged in service and sales occupations in comparison to only 19% of their male counterparts [20]. The main female service and activities were trading in the market (37%) or queueing for water (31%), which were generally accomplished either before the sunrise (73%) or after the sunset (63%) (data not shown). This is probably the reason why more women (61%) than men (37%) with malaria infection step-out into open spaces earlier than 6 am, when the risk of mosquito bites is considered higher.

Notably, the sociocultural practices of interviewees appear to be the main reasons for mosquito exposure, among both males and females, as both tend to spend time in open spaces after the sunset. A similar finding was described in a study conducted in Tanzania, where the men’s habit of spending time outdoors drinking alcohol and watching television was linked to malarial risk [15].

Ghana has a long history of using mass media and other communication channels to educate the population about malaria [21]. The Ashanti Region has the highest rate of household ownership of information and communication technology (ICT) equipment according to the MICS 6. These ICT features could explain the high rate of malaria awareness found among our study population, mainly attributable to radio and television advertisements.

Differently from that reported in the MICS 6, 57% of households in Ghana own at least one ITN [20]. In line with other studies conducted in sub-Saharan African countries showing a similar trend in ITN use [22,23], our survey revealed that only 39.6% of cases own an ITN. Similarly, this study shows a very low use of ITN among highly educated healthcare professionals. Low ITN use, especially in highly urbanized settings, could be explained by the adoption of different malaria-prevention measures; for example, wealthier households tended to have better access to housing improvements, like window screens and closed eaves, which reduce exposure to indoor mosquito bites [24]. Having a decreased perception of vulnerability to malaria also resulted into decreased net use [25]. Another recent study also found that net ownership and use vary widely across sociodemographic groups within and across countries [22].

Both IDIs and FGDs indicated that the interviewees find ITNs uncomfortable, referring to heat and claustrophobic feelings. Both male and female interviewees in Fankyenebra complained because of the four-square-net, the type of ITN generally available in pharmacies and shops or provided free of charge during government malaria-prevention campaigns, cannot be easily installed in their small houses. Poor housing is common in these regions, where most people live in uncompleted buildings with no indoor toilets, potable water, electricity or waste disposal facilities, the most common toilet facility is the public toilet used by 41% of the population, and also used by two of five urban households in the country [26]. In urban areas, 30% of households are disposing liquid waste into the gutter. Therefore, often the words “hygiene”, “cleanliness”, “waste”, and “environment” were mentioned as the most favorable habitats for mosquito breeding during all FGDs.

Indeed, the FGDs interviewees mentioned precarious conditions of dwellings as a source of malaria infection, while Darko male respondents proposed that women get more malaria because they more frequently use public toilets, which are potential areas of mosquito activity due to septic tanks within communities acting as potential vector breeding sites [27]. Noteworthy, misconceptions about being more susceptible to malaria, for example by eating oily foods or not having good personal hygiene, reported by the community members in our study were very similar to those reported in southern Ghana about twenty years ago [28].

The preference for home treatment and self-initiated medication mostly by men (43%) is highlighted by the quantitative approach as much as by the FGDs. Herbal remedies were also identified as a preferred method of malaria treatment in other research conducted in Kintampo (Ghana) in 2016 [29]. The employment of mixed methods, used for this study, produced multiple insights concerning traditional beliefs about malaria and herbal preparations, the latter of which are also used elsewhere in Africa [30,31]. In contrast, a different attitude towards sickness in children was reported, mostly relying on formal health facilities instead of self-initiated medication. The same feature was described for another study conducted in the Upper West Region [32] where only 3% of children with malaria were treated at home using herbs and leftover drugs.

The quantitative measure of PD did not reveal gendered differences in our cohort, which is consistent with other investigations conducted in Ghana [33,34] and may be due to the widespread use of personal antimalarial measures, greater awareness of malaria as a deadly disease and adequate knowledge about its prevention among the more urbanized and better educated population [33].

Urban environments are less favorable for vectors that have a strong preference for unpolluted waters [7,35], and mosquito dispersal is also limited in urban areas due to higher housing density [36]. Therefore, the low parasite density found in our patients may be attributable to the urban setting where they live.

This study had some limitations, as it was the first research attempt in this area of Kumasi and, specifically, in a recently established healthcare facility and employing a novel research approach. Inevitably, we encountered some challenges. The sample size was relatively small, due to objective difficulties along the enrollment and limited to patients living in the surroundings of HXC. Nevertheless, we are confident that the recruited cases are representative of the population of the area, as shown by the distribution of the sociodemographic characteristics of malaria cases, which are similar to those found in other studies carried out in the country. The main strength of our research is its mixed quantitative-and-qualitative approach, which allowed us to gain new insights into gender and malaria risk factors.

## 6. Conclusions

The mixed-methods approach employed in this study allowed us to identify gender differences in malaria exposure, prevention, and treatment. The patients were studied from a clinical perspective, but risk factors linked to exposure behaviors and preventive measures were also considered. Our data suggest that women are more exposed than men to malarial infection, because the main female house-tasks expose them to mosquito bites during the most dangerous hours. The study also shows a low incidence of ITN use and an increased self-initiated medication among respondents compared to other studies. Women’s use of ITN appears also lower than men, though not to a statistically significant degree.

Moreover, the health literacy (HL), a measure of the ability of people to access and use information to make decisions related to their health [18], was poorer in the female population of our study, confirming the unbalanced education distribution between genders. The investment on community engagement and HL should be the way forward for malaria elimination in LMICs, and also for other infectious diseases all over in the world.

In addition, our study showed how gender, socioeconomic aspects, and cultural attitudes and beliefs could also play a role in the spread of malaria. Given this multifactorial scenario, several mathematical models have been proposed to tackle specific problems related to malaria transmission in a human community, and a promising impact on disease prevention strategy is provided by “agent-based models” [37,38].

The mixed methods approach used in this survey has proved to be valuable for investigating people’s behaviors and awareness, as regards effective malaria prevention measures. Changing people’s behavior and practices is a long journey and will require a deep cultural and political shift, especially in LMICs. As seen for Ebola during the most recent outbreak in West Africa [39] and for HIV in South Africa, community engagement in public health could be the best approach in LMICs. Our study highlights the need to invest in future malaria policies and research tools that are more focused on people’s social and behavioral aspects and not only concentrated on biological or clinical factors. Malaria’s role in human social existence is an intricate and compelling story: each chapter should be considered in order to understand the entire plot.

## Figures and Tables

**Table 1:** Sociodemographic characteristics of malaria patients (cases).

	Male	Female	Total
	n = 47 (%)	n = 77 (%)	n = 124 (%)
Age (years)			
18–29	19 (40.4)	29 (37.7)	48 (38.7)
30–49	21 (44.7)	32 (41.6)	53 (42.7)
>50	7 (14.9)	16 (20.8)	23 (18.6)
Place of Residence			
catchment area	16 (34.0)	33 (42.9)	49 (39.5)
outside catchment area	31 (66.0)	44 (57.1)	75 (60.5)
Education *			
no education	2 (4.3)	14 (18.2)	16 (12.9)
educated	45 (95.7)	63 (81.2)	108 (87.1)
school attendance			
primary	13 (28.9)	31 (49.2)	44 (40.8)
secondary	24 (53.3)	24 (38.1)	48 (44.4)
higher	8 (17.8)	8 (12.7)	16 (14.8)
Literacy *			
poor	10 (21.3)	38 (49.4)	48 (38.7)
good	35 (74.5)	36 (46.8)	71 (57.3)
visually impaired	2 (4.3)	3 (3.9)	5 (4.0)
Occupation *			
skilled labors	7 (14.9)	7 (9.1)	14 (11.3)
students	12 (25.5)	8 (10.4)	20 (16.1)
arts and crafts	3 (6.4)	14 (18.2)	17 (13.7)
cooking and catering	2 (4.3)	6 (7.8)	8 (6.5)
traders and retailers	5 (10.7)	30 (39.0)	35 (28.2)
transport	5 (10.6)	-	5 (4.0)
farmers	5 (10.6)	2 (2.6)	7 (5.7)
unskilled labors	4 (8.5)	4 (5.2)	8 (6.5)
others	4 (8.5)	6 (7.8)	10 (8.1)

* *p* < 0.05. Note: Occupations: Skilled labors include (engineering and construction, healthcare and teaching); students; arts and crafts include (photographer, beading and decoration, stylish and fashion, sewing jobs); cooking and catering; traders and retailers; transport; farmers; unskilled labors include (cleaners, porters, watchmen, and receptionist); others (unemployed, retired, public sector jobs).

**Table 2:** Laboratory parameters of malaria patients (cases).

	Male		Female	
	n	Mean	n	Mean
Parasite density (*p*/µL) *	47	2344.4 (1374.3–3999.1)	77	1422.6 (848.1–2386.5)
Hemoglobin (g/dL)	47	14.1 (±1.2)	70	12.0 (±1.4)
Glycemia (mmol/L)	44	6.2 (±1.8)	67	6.9 (±3.6)

* Geometric mean and range, n = number.

**Table 3:** Malaria awareness, exposure behaviors and preventive measures among cases (male vs. female).

	Male	Female	Total
	n = 47 (%)	n = 77 (%)	n = 124 (%)
Malaria Awareness			
knowledge about malaria	45 (95.7)	75 (97.4)	120 (96.8)
advertisements (television, radio, poster/billboard, newspapers)	43 (91.5)	71 (92.2)	114 (91.9)
community based approaches (CHWs, community health events, hospital)	13 (27.7)	33 (42.9)	46 (37.1)
anywhere else (school, information center, etc…)	13 (27.7)	26 (33.8)	39 (31.5)
Malaria medication self-initiation	20 (42.6)	20 (26.0)	40 (32.3)
Malaria preventive measures			
mosquito coil	14 (29.8)	32 (41.6)	46 (37.1)
indoor spray	19 (40.4)	29 (37.7)	48 (38.7)
windows screen	27 (57.5)	40 (52.0)	67 (54.0)
door screen	9 (19.2)	18 (23.3)	27 (21.8)
ITN ownership	20 (42.6)	29 (37.7)	49 (39.6)
ITN use			
always	11 (55.0)	13 (44.8)	24 (49.0)
sometimes-never	9 (45.0)	16 (55.2)	25 (51.0)
ITN maintenance			
fully intact	17 (85.0)	19 (65.5)	36 (73.5)
damaged	3 (15.0)	10 (34.5)	13 (26.5)
Malaria exposure behaviors			
first step-out in open space			
earlier than 6 a.m.	17 (36.2)	46 (59.7)	63 (52.1)
later than 6 a.m.	29 (61.7)	29 (37.7)	58 (47.9)
unknown	1 (2.1)	2 (2.6)	3 (2.4)
going in open space, during the night hours (10 p.m.–6 a.m.)	33 (70.2)	43 (55.9)	76 (61.3)
socializing in open space after the sun set			
often	36 (76.6)	56 (72.7)	92 (74.2)
sometimes–never	11 (23.4)	21 (27.3)	32 (25.8)
last month gathering and sleeping outside	25 (53.2)	38 (49.4)	63 (50.8)

ITN, insecticide treated nets; CHWs, community health workers.

## Data Availability

The datasets used during the current study are available from the corresponding author on reasonable request. The Authors are responsible for the views contained in this article and for opinions expressed therein, which are not necessarily those of UNESCO and do not commit the Organization.
